# Improved longevity of actomyosin in vitro motility assays for sustainable lab-on-a-chip applications

**DOI:** 10.1038/s41598-024-73457-x

**Published:** 2024-10-01

**Authors:** Andreas Melbacke, Aseem Salhotra, Marko Ušaj, Alf Månsson

**Affiliations:** Department of Chemistry and Biomedical Sciences, Linnaeus University, 39182 Kalmar, Sweden

**Keywords:** In vitro motility assay, Actomyosin, Lab-on-a-chip, Biosensing, Network based biocomputation, Microfluidics, Biochemistry, Biophysics, Biotechnology, Nanoscience and technology

## Abstract

In the in vitro motility assay (IVMA), actin filaments are observed while propelled by surface-adsorbed myosin motor fragments such as heavy meromyosin (HMM). In addition to fundamental studies, the IVMA is the basis for a range of lab-on-a-chip applications, e.g. transport of cargoes in nanofabricated channels in nanoseparation/biosensing or the solution of combinatorial mathematical problems in network-based biocomputation. In these applications, prolonged myosin function is critical as is the potential to repeatedly exchange experimental solutions without functional deterioration. We here elucidate key factors of importance in these regards. Our findings support a hypothesis that early deterioration in the IVMA is primarily due to oxygen entrance into in vitro motility assay flow cells. In the presence of a typically used oxygen scavenger mixture (glucose oxidase, glucose, and catalase), this leads to pH reduction by a glucose oxidase-catalyzed reaction between glucose and oxygen but also contributes to functional deterioration by other mechanisms. Our studies further demonstrate challenges associated with evaporation and loss of actin filaments with time. However, over 8 h at 21–26 °C, there is no significant surface desorption or denaturation of HMM if solutions are exchanged manually every 30 min. We arrive at an optimized protocol with repeated exchange of carefully degassed assay solution of 45 mM ionic strength, at 30 min intervals. This is sufficient to maintain the high-quality function in an IVMA over 8 h at 21–26 °C, provided that fresh actin filaments are re-supplied in connection with each assay solution exchange. Finally, we demonstrate adaptation to a microfluidic platform and identify challenges that remain to be solved for real lab-on-a-chip applications.

**Supplementary Information:**

The online version contains supplementary material available at 10.1038/s41598-024-73457-x.

## Introduction

Biological molecular motors of the myosin superfamily are powered by the turnover of ATP to produce force and motion by interacting with actin filaments for a range of cellular functions^1^. This includes muscle contraction, non-muscle cell motility, and intracellular transport. One way to study these motors is by using the in vitro motility assay^2–4^ where the myosin motors, or myosin motor fragments, are immobilized on appropriately treated surfaces that are assembled into flow cells to allow convenient solution exchange. The myosin-induced propulsion of fluorescence-labelled actin filaments is then observed using a fluorescence microscope. This allows the derivation of a range of functional data that has been instrumental in understanding fundamental aspects of actomyosin function^5–10^, including velocity vs. [MgATP]^4,11^, effects of frictional loads^12,13^, troponin-tropomyosin mediated Ca^2+^-activation^14,15^ and actin filament flexural rigidity^16^. In the mentioned studies, it is generally sufficient to observe the motor-propelled filaments for short periods, say in the range 10–30 min (depending on myosin isoform) with limited requirements for prolonged function. However, preservation of function for much longer periods is desirable in certain cases. This includes high-throughput screening of drugs or testing of a range of interventions where it may be of interest (both for time saving and saving of precious protein samples) to test several different drugs, drug concentrations or different interventions, using one motility assay flow cell. Such experiments are of relevance in view of the emerging interest of various myosin motors as drug targets^17^, e.g. in hypertrophic cardiomyopathy^18^ heart failure^19^, skeletal muscle related disorders^20^, cancer^21,22^, malaria ^23–25^ etc. Other applications where extended longevity is essential is the use of myosin motors in nanotechnological applications^26–29^. The latter include transport of analyte molecules in nanoseparation and/or biosensing (e.g. in diagnostics)^30–33^ where the molecular motor driven transport substitutes conventional microfluidics/nanofluidics systems. Such systems allow unprecedented miniaturization and the independence of bulky and expensive accessory equipment such as high-pressure pumps required to drive nanofluidic transport. Network based biocomputation^34–37^, where exploration of large, nanofabricated networks by myosin propelled actin filaments solves complex mathematical problems, is a related application where prolonged motor function is important. The reason is that effective computation requires a very large number of exploring actin filaments. This can be achieved by adding more filaments and use many networks in parallel but also by re-using the filaments many times. The latter strategy would be much more powerful, the longer the myosin driven filament transport is sustained.

In a recent study^38^, we reported appreciably prolonged actomyosin motor function in the in vitro motility assay, either by sealing the flow cells to minimize air access or by substituting glucose oxidase for pyranose oxidase in the glucose oxidase – glucose – catalase oxygen (GOC) scavenger system. Both these interventions are believed to be effective in prolonging myosin function by preventing the formation of gluconic acid. The latter substance forms when glucose oxidase catalyzes the reaction between oxygen and glucose. In contrast, pyranose oxidase does not catalyze the formation of any similar acidic substance^39^ and sealing of the flow cell would be expected to prevent gluconic acid formation by limiting the oxygen content. However, both sealing of flow cells and exchange to pyranose oxidase come with disadvantages. Thus, pyranose oxidase is appreciably more expensive (almost 100-fold) than glucose oxidase and sealing of the flow cell prevents solution exchange, which is detrimental to drug screening applications and various nanotechnological applications such as the addition of analyte-containing solutions in biosensing or the addition of identity tags in some proposed versions of network based biocomputation^36^. However, if the formation of gluconic acid by GOC is what limits longevity, one would expect that exchange of assay solution at regular intervals would be useful in prolonging motility as it would remove the formed gluconic acid. Additionally, if carefully degassed assay solution is used, exchanges would be expected to lower the oxygen content of the flow cell^38^. Exchange of assay solution, on the other hand, may come with other problems, particularly if the motility assays need to go on for hours and require repeated solution exchanges. This includes possible loss of myosin motor fragments by surface desorption during each solution exchange^40–42^, motor denaturation, evaporation effects between exchanges and/or loss of actin filaments.

**Fig. 1:**
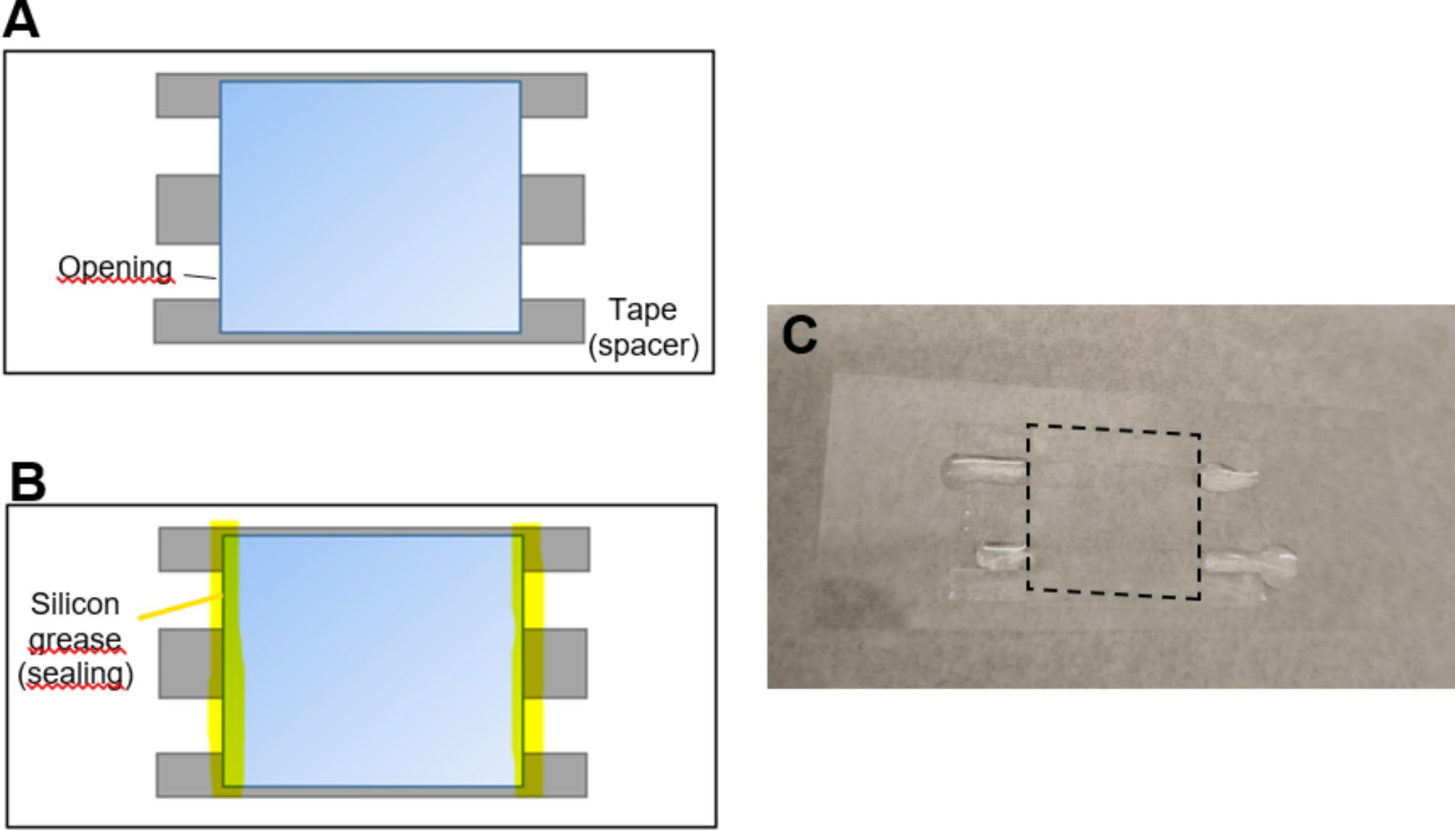
Assembled conventional flow cells. (**A**) Schematic drawing of open non-sealed flow cells with access to fluid via left and right openings of each cell. (**B**) Schematic drawing for flow cell assembly after addition of silicone vacuum grease to seal the cells. (**C**) Photography of assembled flow cell (with roof coverslip indicated by dashed lines) with droplets of fluid outside flow cell openings to avoid evaporation of fluid from the flow cell itself.

Here, we tested the above hypotheses by several specific analyses of pH, oxygen content etc. and by investigating if we could preserve motility for extended times by regularly exchanging the GOC-containing assay solution without adding additional heavy meromyosin motor (HMM) fragments. In most experiments we used conventional^3^ in vitro motility assay flow cells (Fig. 1) both for testing the effects of different factors and for developing an optimized protocol for use in applications. Our results support the idea that solution exchange is of critical importance for prolonged motility if inert atmosphere chambers are not used, primarily to prevent negative effects of oxygen, such as the mentioned lowering of pH with GOC-containing assay solution. Our results also show that it is important to supply new actin filaments upon solution exchange. However, but we find no evidence for appreciable HMM inactivation (e.g. due to denaturation or oxidation) or desorption from the flow cell surfaces even with up to eight hours of experimentation. We also adapted our protocol and described new challenges of a simple low-pressure microfluidics platform where we found that motility was maintained for several hours under continuous assay solution flow. In summary, this work elucidates factors that limit prolonged in vitro motility assays with maintained high motility quality. Moreover, it reports viable protocols for prolonged (hours–day) conventional motility assays for testing several drugs or interventions. We also show how these protocols can be extended into a simple microfluidic system. However, also in this process we found that challenges remain to solve before realizing lab-on-a-chip applications such as biosensing and network based biocomputation with motility consistently maintained for more than 5 h.

## Results

### pH and motility vs time – effects of sealing of conventional flow cell

**Fig. 2:**
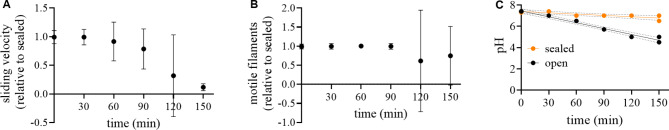
Time-dependent decrease of motility quality is associated with a drop in pH in open flow cells. (**A**) Relative sliding velocity in open flow cells (velocity in open cell/velocity in closed cell) vs. time after adding assay solution. Each data point represents mean ± 95% CI based on three independent experiments except point at 150 min, representing two experiments. The experiments were performed in a paired design (open vs. closed cell) on three different days with different stock solutions including myosin and actin batches and partly different personnel. Data for individual experiments in open and sealed flow cells are presented in Fig. S2. (**B**) Relative fraction of motile filaments (fraction in open cell/fraction in closed cell) vs. time as in (**A**). Each data point is given as mean ± 95% CI based on three or two independent experiments (same as in **A**). (**C**) pH vs. time for open (black) and sealed (orange) flow cells, estimated using pH paper (Figs. S3, S4). The solid lines represent linear regression curves fitted to the underlying data and given with 95% CI (dotted lines). pH was 7.4 at start of all three experiments. Data from one experiment at times 30–120 min (other flow cell than motility experiment) and from two other experiments at time 150 min (same flow cells as motility). Overall, note that the actin filament sliding velocity in open chambers tended to decline after 60 min but with a similar fraction of motile filaments up to at least 90 min. After that, associated with a pH drop below ∼6 in the measurements in (**C**), a clear decrease in motility quality is demonstrated both by the decline in velocity and by the variable fraction of motile filaments. Particularly, the 95% CI for the fractional velocity (open vs. sealed) at time 150 min is clearly below the value at time 0 min (1.0), reflecting a statistically significant difference (see also Fig. S2).

We hypothesized above that the decline of motility quality (velocity and fraction of motile filaments) with time, in open flow cells in motility assays using the GOC oxygen scavenger system^38^, is primarily due to lowered pH due to accumulation of gluconic acid. First, we corroborated the difference in motility quality between open and sealed flow cells using a paired experimental design where experiments in open and closed flow cells was performed in parallel on the same day by a given experimenter using the same solutions as well as actin and myosin batches. (Fig. 2). We found that motility quality (velocity and fraction of motile filaments) was almost constant for more than 2 h in sealed flow cells (Fig. S2), consistent with unchanged velocity in such flow cells for up to 4 h in a previous study^38^. In open flow cells, on the other hand, motility significantly deteriorated with time accompanied by a marked decrease in pH. Notably, after 60 min, the pH was reduced from an initial value of 7.4, to ∼6 in the open flow cells whereas pH was only minimally reduced in the sealed flow cell after 120–150 min (Fig. 2C). In previous work, with control of pH in motility assay experiments, a decline of pH to 6 was found to be associated with complete loss of motility^4,43,44^. We attribute the fact that motility was observed for more than 100 min in our experiments (Fig. 2) to a delay in the pH reduction near the center of the flow cell, considering that pH was measured by immersing the pH papers in assay solution at the cell opening. Importantly, the results in Fig. 2 show that the decline in velocity and fraction of motile filaments is negligible for an incubation period of up to 30 min even with open flow cells, supporting the use of time intervals between assay solution exchanges ≤ 30 min. The data in Fig. 2 also show variability between experiments in the open flow cells, particularly at times > 100 min. This variability reflects a decline in motility quality with variable time course of the process in different experiments. We attribute the latter variability to different experimenters as well as different batches of chemicals, proteins, cover-slips, tapes for flow cell spacers, etc.

We were somewhat surprised by the quick acidification of the assay solutions (Fig. 2C) considering that we are performing extensive degassing (~ 40 min at -0.8 bar vacuum) of our major assay buffer component (typical volume of 20 ml) before the experiments. We therefore tested our degassing capabilities and re-oxygenation of degassed solution by using an oxygen meter. This analysis (Fig S5) shows that the lowest oxygen content level is reached already after 20 min of degassing. However, it also shows that the subsequent reoxygenation is rather fast (+ 50% in 1 h) when 20 ml solution is kept in a closed 50 ml centrifuge tube on ice. Addition of N_2_ into the tube, closing the cap and sealing it with parafilm slowed down the reoxygenation. Based on these results, the fast acidification of the assay solution in an open chamber seems fully explained as being produced by the GOC oxygen scavenging system acting on dissolved oxygen.

The oxygen meter-based data in Fig. S5 also imply that assay solution needs to be prepared as soon as possible after the main assay buffer component (LISS) degassing followed by storage in a closed syringe^38,45^, to preserve the low oxygen content during an experimental day.

Velocity (and pH) declines slowly with time also in sealed flow cell (cf. Fig. 2) possibly due to incomplete sealing or leakage of oxygen into the flow cell by other mechanisms. However, calculations^28,38^ suggest that the reduction in velocity is not due to accumulation of the ATP hydrolysis products, inorganic phosphate (Pi) or ADP. Our data below, with > 8 h motility when the assay solution is repeatedly exchanged in conventional flow cells, also show that the small decline in velocity with time is not due to HMM-loss from the surface by desorption or HMM denaturation.

### Towards a standard protocol for conventional flow cells

**Fig. 3:**
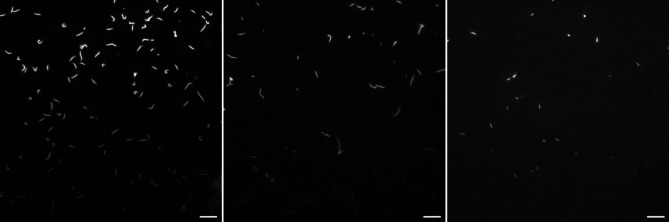
Loss of actin filaments during multiple exchanges of assay solution. Rhodamine phalloidin labeled actin filaments are shown in gray. The images are taken after each consecutive assay solution exchange at 20, 40 and 60 min, from left to right. A visibly lower concentration of actin filaments is seen with each solution exchange. Scale bar, 10 μm.

In an initial experiment, using a60 assay solution (assay solution with 60 mM ionic strength), we found that, with each exchange of assay solution, some actin filaments were washed away (Fig. 3). For the next experiment, actin filaments were therefore re-supplied with each exchange. In this procedure, the old assay solution was first washed away using a wash buffer, then actin filaments were added followed by another wash and, finally, addition of fresh assay solution (a60). In this initial experiment, the modified protocol, including the addition of actin filaments, was found to allow prolonged motility without any appreciable decline in the number of actin filaments.

Another issue that may lead to a decline in velocity and motile fraction with time in open flow cells is evaporation via the flow cell openings causing increased ionic strength in the assay solution over time. If the ionic strength increases above ∼80 mM, filament detachment and/or erratic movement are likely to occur in the absence of methylcellulose. Our analysis in the Supporting Results suggests that effects of this type are likely to appear unless a liquid chamber is used for flow cell storage between motility observations.

**Fig. 4:**
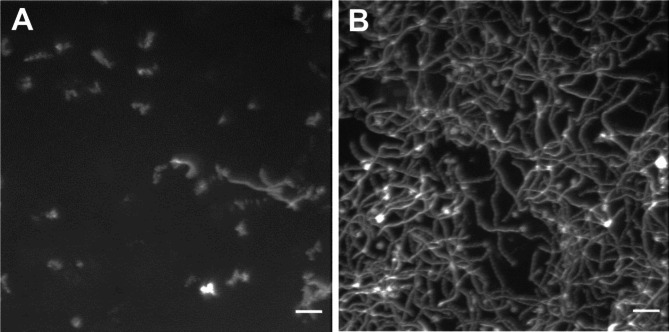
Loss of actin filaments from surface to the fluid phase, compared to good motility. Summed maximum intensity projection (Z-projection in ImageJ) of 20 consecutive grayscale images of Rhodamine phalloidin labelled actin filaments captured at 5 FPS for 4 s. (**A**) A vast majority of the actin filaments were floating in solution and were not attached to the surface as indicated by blurred projection reflecting Brownian motion. (**B**) Attachment of all filaments with good motility quality as indicated by extended paths traced out by a majority of the actin filaments. Image intensities adjusted for clarity. Scale bar, 10 μm.

**Fig. 5:**
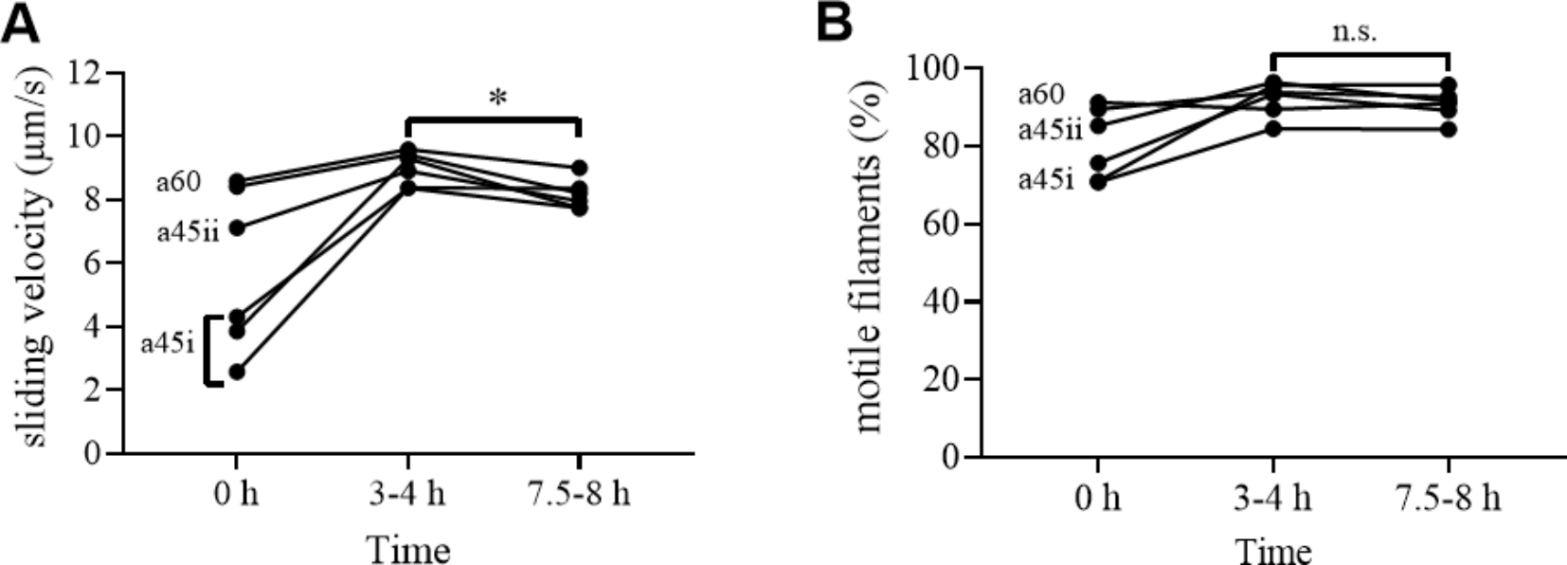
Average gliding velocity and fraction of motile filaments vs. time for 6 different experiments. (**A**) Gliding velocity. (**B**) Fraction of motile filaments. **a60**: protocol using a60 assay solution with storage at 21 °C on wet paper between motility assays (performed at 25 °C) showing results of the 2 successful experiments. Individual data in Fig. S6. **a45i**: protocol using a45 assay solution without objective based temperature control, but storage of the flow cells on the microscope stage between in vitro motility assays, starting with room temperature (21 °C) but with temperature of ∼25 °C after 4 h. Individual data in Fig. S8. **a45ii**: protocol using a45 assay solution with storage of flow cell on the microscope stage between in vitro motility assays with temperature in the range 24–25 °C during the entire experiment. Individual data in Fig. S9. Note, the initial increase in velocity and fraction of motile filaments from 0–4 h in the a45i protocol can be attributed to the increase in temperature. Note further that both velocity and fraction of motile filaments in the a45i protocol reach approximately the same values as in the other protocols at 3–4 h. Note, finally, that whereas the average velocity changes significantly from 4–8 h (*p* = 0.014; *) the mean change is only 0.8 μm/s (approximately 10%) and the fraction of motile filaments does not change significantly (*p* > 0.05; n.s.).

We therefore performed a new set of experiments with storage in the mentioned type of liquid chamber at 21 °C between motility assays and with actin filament addition in connection with solution exchange (see above). The experiments were performed using 4 different flow cells and motility was observed at 24–26 °C in a60 assay solution. In two of the flow cells, motility was lost (cf. Fig. 4a) with actin filaments floating in the solution, whereas the other two flow cells exhibited high-quality motility with constant velocity at 7–10 μm/s and fraction of motile filaments (90%) for 15–16 exchanges (> 7.5–8 h; cf. Fig. 4b). The time evolution of velocity and fraction of motile filaments, for the two successfully tested flow cells is indicated in Fig. S6, with average values given in Fig. 5 together with data obtained under other conditions (see below). We found that sliding velocity and the fraction of motile filaments changed negligibly for these cells over the 7.5–8 h periods studied. This is important because it suggests that neither deterioration of myosin function, loss of actin filaments, nor desorption of myosin from the TMCS surface occurs to any significant degree despite 16 exchange sessions, i.e. >16 × 4 = 64 solution exchange steps, each with 10–20 µl volume and fast flow. The observation of filaments floating in solution in the two unsuccessful experiments using a60 assay solution is consistent with too high ionic strength of the assay solution (> 80 mM) which could result from evaporation (see above). As a precaution, we therefore decided to reduce the ionic strength in our assay solution to 45 mM (a45 solution). As observed previously^4,46^, this had minimal effect, in itself, on motility quality (velocity and fraction of motile filaments; Fig. S7) giving only a small decrease in velocity. Importantly, however, the risk of deleterious effects of increased ionic strength due to evaporation would be greatly reduced. Thus, even the highest degree of evaporation during a 30 min period (see above) would not increase the ionic strength of this solution above 75 mM. In the following, we therefore decided to use an assay solution with ionic strength of 45 mM (a45 solution) unless otherwise stated.

## Protocol for improved motility longevity using conventional flow cells

Our analysis above suggests a standard protocol for studies using conventional flow cells. This would encompass exchange of assay solution every 30 min accompanied by addition of new actin filaments in association with each exchange and use of an assay solution of 45 mM ionic strength. However, in contrast to the experiments above, with storage of the flow cells outside the microscope stage (on wet paper to minimize evaporation), continuously on-going long-term functional assays and nanotechnological applications would most conveniently be performed with the flow cell kept continuously on the microscope stage. Because experiments using conventional flow cells are also useful in nanotechnological applications^30,34,37^, we tested protocols that allow such a procedure.

First, (i) we kept the flow cell continuously on the microscope stage without the objective temperature control turned on. Second, (ii) we kept the flow cell continuously on the stage with the objective temperature control ring turned on to keep temperature constant at 24–26 °C. In these experiments, storage of the flow cell on wet paper in between exchanges was impossible for practical reasons. The most important result from these experiments is that, in all cases, (4 experiments in total), high-quality motile function was maintained for at least 8 h. The averages for velocity and fraction of motile filaments for each of the experiments are plotted in Fig. 5 together with the data with 7.5–8 h successful motility in a60 solution (see above; Fig. S6). The averages for velocity and fraction of motile filaments, respectively, are based on measurements of 15 filaments and 3 regions of interest per time point in each experiment (Figs. S6, S7–S9). Importantly, the results suggest very well-maintained motility in conventional motility assay flow cells over an observation period of 8 h at 21–25 °C using our standard exchange protocol. First, both velocities and fraction of motile filaments at 4–8 h for all protocols in Fig. 5 are similar to the velocities and fraction of motile filaments for the protocols at time 0 h where temperature was 25 °C. Second, there was only a minimal reduction (∼10%; *p* = 0.014) in gliding velocity and no change in fraction of motile filaments between the 4 and 8 h time points in Fig. 5 when all experiments are considered together. The initial quite substantial increase in velocity observed in three experiments (protocol a45i in Fig. 5) is due to increased temperature. Whereas the intention was to perform the experiments at a constant ambient temperature, with the objective temperature control system switched off, temperature actually increased with time from 21 °C at the start of the experiment to 25.5 °C at the end. We consider this effect further at the end of the Supporting Information. However, this complication aside, the important take-home messages from Fig. 5 is that high-quality motility (velocity and fraction of motile filaments) is possible to maintain for at least 8 h using our standard exchange and assay protocol.

The beneficial result above were associated with some complexities. Thus, after a certain time (starting after approximately 5–6 h), the flow cells became increasingly filled with air bubbles but there were still regions with full functionality as studied in Fig. 5. The origin of the bubbles was unclear, but they might be due to leakage under the tape-spacers, due to loss of stickiness after prolonged contact with the liquid. We did not further pursue this issue because it would anyway be inconvenient to perform the experiments for longer periods with manual fluid exchange in conventional flow cells. Moreover, we anticipated that switching to a pump-driven microfluidics system would remove the bubble issue. Indeed, this turned out to be the case (see below) but unfortunately without eliminating the emergence of motility non-uniformity over time.

## Adaptation for a microfluidics platform

**Fig. 6:**
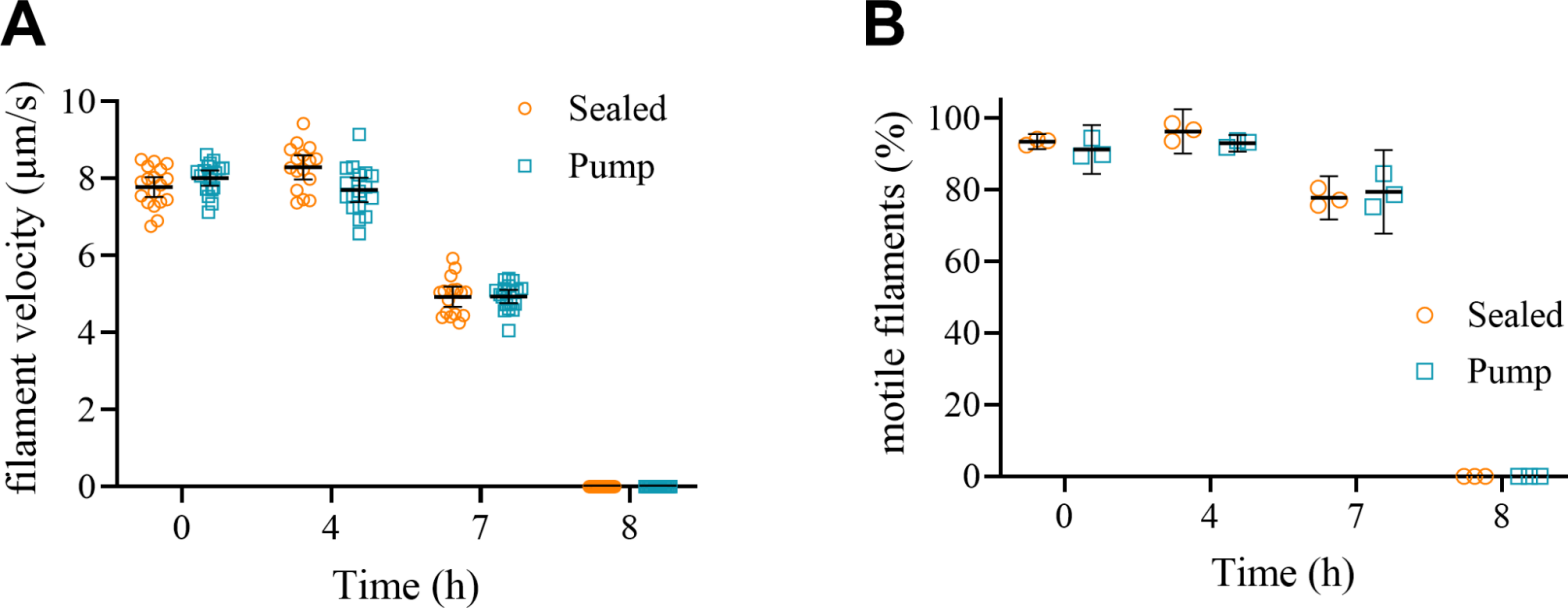
Gliding velocities and fraction of motile filaments vs. time using a microfluidic platform. (**A**) Gliding velocities vs. time after the addition of assay solution with the microfluidic chamber sealed (orange) or open (magenta). Each time point is represented by mean ± 95% CI based on measurements on 15–18 filaments. (**B**) Fraction of motile filaments vs. time in 3 regions of interest of the same flow cells as in A, either sealed (orange) or open (magenta). Each time point is represented by mean ± 95% CI. T = 24–25 °C.

We adapted the standard protocol developed above to a simple low-pressure microfluidics platform. This simple system is composed of a polymer block (IBIDI sticky-Slide VI 0.4) fitted with 6 chambers of small volume 30 µl) to which a TMCS derivatized coverslip was firmly attached. Previously, we have used this system with manual solution exchange following the final addition of assay solution and sealing of inlets with parafilm^38^. This time, however, one of the microfluidics chambers was instead connected to a syringe pump for control of fluid flow (Fig. S10A)^38^. Experiments were performed essentially with the conditions mirrored from the protocol above with the following modification. We supplemented an a60 assay solution with extra 2 nM rhodamine-phalloidin-labeled actin filaments to counteract the effect of actin filament depletion during continuous pump operation. Like above, we kept the microfluidic assembly continuously on stage in contact with the objective and the temperature control ring was turned on as depicted in Fig. S10A. As the microfluidic chamber volume was 30 µl, the initial pumping speed was set to 1 µl/min to effectively replace the entire assay solution volume in 30 min as suggested suitable based on manual solution exchange experiments. However, this led to a low quality of motility. A protocol where the pumping speed was reduced to 0.2 µl/min was consequently adopted. In parallel, we also performed motility in sealed microfluidics chambers where the inlets were sealed with vacuum grease (Fig. S10A). This set-up essentially mimics our standard sealed chamber conditions with the difference of 6–12 times higher volume of assay solution trapped outside the chamber in the channel inlets (in total ~ 120 µl). The results of the sealed chamber were as expected, reaching at least 4 h of high-quality motility (Fig. 6, Fig. S10B). This exceeds results from our previous attempts using such microfluidic systems and experimental conditions with the GOC oxygen scavenger system^38^. However, we had expected that continuous assay solution exchange via pump action, would improve and/or prolong motility beyond what was observed in the sealed chamber. Surprisingly, this was not the case (Fig. 6, Fig. S10C). Other qualitative observations were that in sealed conditions there was evident elongation of actin filaments reaching maximum at 3–4 h with subsequent shortening of the filaments for the rest of the experiments (up to 8 h). A similar behavior was absent from pump-driven chambers.

## Discussion

### Key findings, their interpretations and relationship to earlier studies

We have demonstrated that high quality motility with a similar fraction of motile filaments and velocity reduction of ≤ 10%, can be maintained for more than 8 h in conventional in vitro motility assay flow cells, using a simple protocol for repeated exchange of assay solution. This is up to eight times more than for open flow cells without exchange (^38^ and Fig. 2). It is also more than double the longevity previously^38^ achieved with GOC as oxygen scavenger despite the use of sealed flow cells.

Naturally, it has been realized for a long time, that there are several critical factors in preserving good motility quality^2,3,47^. However, preservation of motility over several hours has not been in focus because most conventional motility assays in basic research usually study a given flow cell only briefly (< 30 min). When earlier studies^38,48–50^ have considered long-term function they have had various biotechnological applications of the myosin motor system in focus.

The main motivation of the present paper is to better understand the factors that reduce motility quality over time, with the goal to increase the longevity for hours without resorting to advanced, expensive or specialized custom-built equipment. In this regard we considered several factors: oxygen entrance into flow cells causing pH reduction and oxidation of proteins, evaporation leading to increased ionic strength or drying, actin filament loss, HMM desorption or denaturation and temperature variations.

First, the observed favorable effects of the sealing of flow cells (Fig. 2) and exchange of assay solution are in accordance with the idea that oxygen entrance into the cells is a main reason for the decline in motility with time in open flow cells. Moreover, our finding of reduced pH in the open flow cells supports the idea that an appreciable part of this effect is due to accumulation of gluconic acid. This is in agreement with previous data^38^ showing favorable effects of an exchange from glucose oxidase to pyranose oxidase in the oxygen scavenger mixture. Phototoxicity might also contribute to long-term deterioration of function^51^ by mechanisms partly akin to those of molecular oxygen by generating free radicals. However, the total level of illumination in the present experiment was presumably too low to have any major effect in this regard, possibly with the exception of the experiments with microfluidics (see below).

Second, evaporation may compromise long-term function in flow cells that are left open to allow solution exchange. Whereas we demonstrated that a simple humid storage chamber counteracts the evaporation effect, the use of such a chamber is generally not practically feasible. However, importantly, we demonstrated that repeated exchange of assay solution at 30 min intervals in conventional flow cells largely overcome the problem, particularly when using assay solutions of ionic strength below 50 mM.

Third, our investigations demonstrated the need to add new actin filaments or somehow prevent their detachment from surfaces upon solution exchange both when using conventional flow cells and microfluidics systems.

Fourth, importantly, repeated solution exchanges did not cause any severe degree of desorption of HMM motor fragments from the surface. This is somewhat contrary to previous observations from both in vitro motility assays ^52^ and studies using total internal reflection fluorescence TIRF spectroscopy^41,53^ suggesting partial desorption. On the other hand, quite a substantial decrease of the HMM surface density would be required for loss of motility and even decrease in sliding velocity. Thus, using a similar assay solution as here, without methylcellulose, Toyoshima et al.^6^ found a decrease in velocity and loss of motility first when the HMM surface density on nitrocellulose was reduced down to 500–1000 molecules µm^−2^. This critical HMM surface density should be compared to a surface density of 5000–7000 µm^2^ expected on TMCS derivatized surfaces immediately after HMM incubation under our conditions^40,41,53^.

Fifth, with regard to possible denaturation of HMM with time, it is of interest to note that despite rather strong adsorption forces, there is no evidence for myosin head unfolding, considering the maintained high velocities and high fraction of motile filaments for more than 8 h. These results provide strong evidence for very limited myosin inactivation by denaturation or other mechanisms e.g. oxidation, if such inactivation renders the myosin heads ATP-insensitive and rigor-like. This follows from evidence that as little as 1–10% of inactive HMM heads of this type would be sufficient to completely block actin filament sliding^54–56^. Moreover, computational modelling suggests a monotonous decrease in velocity for an increased fraction of inactive heads in the 0–10% range^56^. This applies on hard flat surfaces, as used here, whereas the relationship may be more complex with HMM adsorption on soft polymers such as nitrocellulose^56,57^.

The capacity of HMM to resist unfolding upon adsorption to TMCS, is supported by observation of motility in conventional flow cells, after HMM has been kept in a refrigerator (4 °C) for up to 14 days^49,58^ or in a freezer (−20 °C) for more than one month^49^. Moreover, we demonstrated recently^38^ that motility, driven by TMCS-adsorbed HMM, could be preserved at room temperature for up to 23 h if pyranose oxidase was used in the oxygen scavenger mixture (instead of glucose oxidase). It is worth noting that in those experiments, despite being performed in microfluidic cells with minimal air access, motility was maintained for less than 4 h if glucose oxidase was used in the oxygen scavenger mixture. These results, together with our presently demonstrated effectiveness of the exchange protocol, again strongly support the idea that the motility decline with time in the presence of glucose oxidase is primarily attributed to accumulation of gluconic acid, leading to reduced pH. The appreciable effectiveness of the exchange protocol is emphasized by the fact that we achieved high velocity and fraction of motile filaments for at least 8 h without using blocking actin (non-fluorescent actin at 1 µM concentration usually added to block non-functional heads). This procedure was found essential in the previous study^38^ where motile function was maintained for 23 h in microfluidic chambers using pyranose oxidase instead of glucose oxidase. In that study, we hypothesized that the blocking actin counteracts surface induced denaturation^38^. It seems unlikely that our exchange protocol should reduce the risk of denaturation. Yet, it seems to have beneficial effects beyond preventing accumulation of gluconic acid. An obvious candidate in this regard is reduced oxidation of myosin due to a lowered average oxygen content.

### Relevance for various types of studies in nanotechnology and otherwise

Our results demonstrate that long-term experiments with repeated exchanges of assay solution are possible using conventional in vitro motility assay flow cells and assay solutions with glucose oxidase in the oxygen scavenger mixture rather than pyranose oxidase. This is of interest in itself as it demonstrates an expanded usefulness of the conventional approach for performing in vitro motility assays. The findings are also important because, thus far, most nanotechnological applications of the actomyosin system (e.g.^30,32–34,37^) have relied on the use of conventional in vitro motility assay flow cells.

We tested the in vitro motility assays with the use of TMCS derivatized glass instead of nitrocellulose coated glass surfaces, more commonly employed for these purposes in research labs. However, we have shown previously^26,42,58^ that TMCS derivatized surfaces (glass and smooth SiO_2_) have a number of advantages over nitrocellulose coated surfaces for conventional in vitro motility assays. Moreover, unlike nitrocellulose, TMCS-surfaces are readily nanopatterned which is a requirement for the nanotechnological applications in focus of our attention. Finally, whereas functionalization of glass surfaces with TMCS is somewhat more complicated than nitrocellulose coating of cover slips, the procedure can be readily expanded to silanize a large number of surfaces in parallel and the silanized surfaces may be stored for prolonged periods. With the aim to expand the use of TMCS derivatized surfaces we describe all these procedures in appreciable detail in the Methods.

One might argue that the addition of new actin filaments to the assay solution during an experiment to compensate for lost filaments does not solve key problems in nanotechnological applications. This includes loss of filaments with cargoes in biosensing applications^30^ and introduction of errors in computation systems^34,37^, if new actin filaments enter computational channels in random orientation. However, by adding filaments with specific analyte binding molecules (e.g. antibodies) to the assay solution, the total number of filaments with cargoes would be kept high in the actual device. Moreover, with usual device design ^27,28,30,34,37^, the added filaments land on the surface only in large open areas for motility (loading zones) where cargo is captured in biosensing devices^30^ and where filaments are loaded for biocomputation^34,35,37^. Importantly, we have shown^34,52^ that actin filaments only rarely enter the nanoscale channels directly from solution, minimizing any problem with randomly oriented filaments. The filaments that land in the loading zones are propelled from these zones unidirectionally along the nanochannels^34,52^ towards a trapping zone (biosensing) or read-out zone (biocomputation).

Whereas conventional custom made flow cells apparently work for long-term experiments and solution exchanges, many experiments both for high throughput and high content screening, would benefit from the use of microfluidics platforms. We have previously tested one such platform^38^ that should be useful for both fundamental science, drug discovery or nanotechnology. Here we have expanded this to use of an automated pump allowing well-controlled continous and steady flow rate of assay solution. In principle this should allow in vitro motility assay studies with continuous slow exchange of assay solution intervened by addition of drugs of interest etc. Furthermore one would expect to overcome several challenges associated with the use of conventional custom made flow cells and manual exchange of assay solutions: (i) evaporation effects (due to a completely sealed system except the microfluidic fluid inlets and outlets), (ii) oxygen effects (due to sealing and continuous flow of fresh degassed assay solution) e.g. formation of gluconic acid and protein oxidation, (iii) eventual depletion of MgATP and accumulation of products MgADP and inorganic phosphate, (iv) human errors of any sort in connection with exchanges, (v) high work load.

By overcoming the challenges above by using a microfluidic platform one would expect motility to run appreciably longer than 8 h as demonstrated with manual assay solution exchanges and conventional flow cells. However, from Fig. 6 it is clear that our expectations were not fulfilled. While a number of the challenges (i–v, above) were reasonably addressed, the longevity of the IVMA could not be maintained beyond 8 h. In fact the quality of motility started declining after 4 h and ceased at 8 h, effectively the same as for sealed microfluidic chamber run in paralell. The reasons for that are at the current stage not completely known. From our attempts it seems that continuous flow of the assay solution in the motility chamber does not support long term motility. In our first attempts we tried to mimic the manual exchange protocol of replacing the entire chamber volume with fresh assay solution every 30 min. However, the performance of the motility assay was unsatisfactory, ranging even below that in the sealed chamber run in parallel. Reducing the flow rate 5-fold, improved the motility quality, however not beyond that of the sealed chamber. It could well be that at such conditions the assay solution replacement is just too slow, effectively resulting in sealed-chamber-like conditions. The possibility also exists that the negative effect of continuous assay solution flow could be related to promotion of HMM desorption from the surface or via some other unknown process. A discrete but complete assay solution exchange with programable pump every 30 min may be a better alternative in this regard and consistent with manual actions. Another reason for possible detachment of myosin motors could be the fact that the IBIDI sticky-Slide used as a basis for the microfluidics chamber is made of plastic which could promote further loss of myosin motors from the glass surface via HMM adsorption to the plastic itself. A low protein binding plastic or microfluidic chamber made entirely of glass could represent a better alternative. There is also the possibility of long-term toxicity of the plastic or the adhesive used to attach the cover-slip as has previously been observed with some types of tape used to produce conventional flow cells^38^. Another challenge to address in the future is deteriorating contrast of moving filaments and in general low quality of recordings after prolonged time of observation. Multiple reasons could be responsible for that. Beside general photobleaching (as we did not perform complete wash and replacements of actin filaments as in manual protocol) the introduction of extra 2 nM of rhodamine-phalloidin labeled actin filaments directly into assay solution could contribute to the issue. Thus, our standard actin filament labeling protocol is performed at actin: dye ratio 1:1.5, effectively containing free rhodamine-phalloidin molecules which could (if not washed away as possible in the manual protocol) start accumulating in the chamber walls thus increasing the fluorescence background levels in the videos. Moreover, such accumulation would also be associated with increased phototoxicity. Possibly such phototoxicity with formation of free radicals could also contribute to the reduction in the number of single actin filaments^59^ and the appearance of large actin aggregates observed at later observation time points (movie S4).

Another reason which could affect the signal in videos from experiments on the microfluidic platform (in comparison to the rest of the paper) is that the microscope illumination source had been permanently changed from mercury lamp to LED based light source when these experiments were performed. Although this can have many positive effects (environment, longevity etc.) it also tends to provide weaker illumination power specifically if comparing mercury lamp peak illumination spectral regions. Despite the listed issues and challenges we are convinced that microfluidic based approach is still the proper approach for IVMA based devices where prolonged motility is required. However, a wide range of methodological aspects, as exemplified above, will need to be tested in future work to allow optimization of the microfluidics based long-term assay. This is clearly outside the scope of this study. As a complement, or possibly alternative to such tests, the use of more advanced microfluidics systems may be considered. Thus, microfluidics integrated with semipermeable membranes^60^ could enable medium exchange without shear, preventing loss of actin and HMM without compromising the capability of high quality imaging. For instance it may be possible to follow the movement of an individual sliding filament (now prevented to detach from the surface) for a long period of time provided that it is also confined within the imaging area by suitably designed nanochannels^52^.

## Sustainability and ethics

We have previously demonstrated that myosin motors can be stored in a functional form for more than 10 years at −80 °C^38^. Together with the increased longevity described here, this will reduce the need for animal material and patient samples with ethical implications. Moreover, the prolonged function together with introduction of highly miniaturized assays, (e.g.^61,62^) will reduce the need also for other material and chemicals for protein purification etc. Thus, one nanostructured chip will be possible to use for appreciably longer time and for many more experiments than before. This is important because the fabrication of such chips is demanding on energy, time and personnel. Another contribution in the latter regard is our recent demonstration^63^ that nanostructured chips can be recycled by simple procedures for use with biomolecular motors. Thus, the present work should be viewed together with several recent developments that will all lead to a situation where the use of molecular motors based nanodevices approach commercial viability (see further discussion in^28^).

## Other perspectives and limitations

Whereas associated with some other drawbacks as mentioned in the Introduction, the longevity of the in vitro motility assay type of experiments may most likely be further improved, possibly extensively by using more advanced and/or more expensive, approaches. This includes: (i) placing the whole experimental system in an inert atmosphere^64^, (ii) exchanging glucose oxidase for pyranose oxidase^38^, (iii) removing oxygen using an polydimethyl-siloxane (PDMS) based microfluidic device that deoxygenates buffers without enzymatic oxygen scavengers^51^ (iv) adding components that inhibit protein unfolding such as, seemingly, blocking actin^38^ and the pharmacological chaperone EMD 57033 (^65^; only in nanotechnological applications not to disturb normal function) and (v) always using as freshly prepared proteins as possible^38^. The latter idea has disadvantages from ethical and economical perspectives, however, as it requires the use of more experimental animals compared to the prolonged use of stored proteins. We showed previously that heayv meromyosin is possible to store up to 10 years at −80° with maintained functionality but obviously somewhat reduced long-term stability^38^.

In the context of microfluidic platforms, a programmable pump with the discrete but complete exchange of assay solution every 30 min, with additional possibilities to switch between different solutions (wash, fresh actin, wash, assay) mimicking our manual protocol may be critical for further improvements. In the case of adding labeled actin filaments directly into the assay solution, the actin: dye labeling ratio should be below stoichiometry in order to prevent accumulation/absorption of free dye species in the microfluidic chamber to maintain good quality of recordings.

Such further optimization and prolongation of motility may be particularly important for running very long-lasting biological computations in network based biocomputation^34–37^, where combinatorial mathematical problems are solved by molecular motor propelled filaments that explore, potentially very large, nanofabricated networks of channels.

The approaches to extend the longevity beyond what was achieved previously^38^ may be possible also for other myosins than the myosin II motor fragments studied here. However, this will require that the surface immobilization mechanism (which may vary, often relying on nitrocellulose and connector molecules such as antibodies) are compatible with repeated exchanges of solutions as described.

## Conclusions

We have elucidated key mechanisms underlying the decline of function of conventional in vitro motility assays with time. With starting points in these insights, we designed solution exchange protocols that reproducibly allow conventional in vitro motility assays as well as a microfluidics-based assay to be run for 4–8 h with minimal deterioration of function. This is of particular importance for applications in nanobiotechnology such as biosensing^28–30^ and, not the least, network based biocomputation^34,36,37^ with implications from both a sustainability and ethics perspective. Moreover, the increased longevity would be valuable for high-content and high-throughput fundamental studies as well as drug screening efforts. These are important, in view of the emergence of various myosin motors as drug targets^17^ in hypertrophic cardiomyopathy^18^ heart failure^19^, skeletal muscle disorders^20^, cancer^21,22^, malaria^23–25^ etc.

## Materials and methods

### Ethical statement

We purified myosin and actin from fast skeletal muscle from a euthanized rabbit, according to a procedure approved by the Regional Ethical Committee for Animal Experiments in Linköping, Sweden (ref. 17088-2020). Before euthanization, the rabbit was anesthetized by an intramuscular injection of 0.25 ml Zoletil (active substances: Zolazepam, 6 mg/kg; Tiletamin, 6 mg/kg och Medetomidin, 0.6 mg/kg). The rabbit was then euthanized by injection of 2 ml of penthobarbital (100 mg/ml) in an ear vein. The Linnaeus University veterinary performed all procedures associated with the euthanization. Myosin and actin were from one given rabbit because our focus here was not on actin and myosin function per se but on the performance of a methodological approach. No in vivo experiments on live animals were performed. In view of the two last sentences the ARRIVE guidelines are not applicable. The work related to use of laboratory animals was performed in accordance with the Animal Welfare act (Swedish law: SFS: 2018:1192) and the guidelines of the Swedish Board of Agriculture that oversees use of laboratory animals in Sweden. The mentioned laws and guidelines are in accordance with the EU-Directive 2010/63/EU on the use of animals for scientific purposes.

### Materials

The chemicals used for these experiments were purchased from Sigma Aldrich Stockholm, Sweden (now Merck), except for Rhodamine Phalloidin that was purchased from Thermo Fisher Scientific (Cat. No.: R415). The thin double-sided adhesive tape was purchased from 3 M (3 M-467MP and 3 M-Scotch dispensered roll). Parafilm-M was purchased from Sigma Aldrich (Cat. No.: P7793). Type-F microscope immersion oil was purchased from Nikon Instruments (Cat. No.: MXA22192). Syringes with 1 ml volume were purchased from B. Braun (Omnifix-F Solo).

### Protein isolation

Myosin and actin were isolated from rabbit skeletal muscle; myosin^66^ from fast leg muscles and actin^67^ from back muscles. Heavy meromyosin (HMM) was prepared from myosin^3^ through limited proteolysis of myosin, using Nα-Tosyl-L-lysine chloromethyl ketone hydrochloride (TLCK) treated chymotrypsin. The protein concentration and purity were evaluated using absorbance spectrophotometry and sodium dodecyl sulphate polyacrylamide gel electrophoresis (SDS-PAGE).

### Solutions for motility assays

A low-ionic strength solution (LISS), degassed as described below, was the main component in all solutions used. LISS has an ionic strength of 15 mM, pH of 7.4 and contains 10 mM 3-(N-morpholino) propanesulfonic acid (MOPS), 1 mM magnesium chloride (MgCl_2_) and 0.1 mM potassium ethylene glycol-bis(β-aminoethyl ether)-N, N,N′,N′-tetraacetic acid (K_2_EGTA). A wash buffer prepared in LISS included 50 mM KCl and 1 mM dithiothreitol (DTT). The assay solution prepared in LISS includes (final concentrations): 45 mM KCl, 10 mM DTT, 1mM magnesium adenosine triphosphate (MgATP), 2.5 mM creatine phosphate (CP) and 0.2 mg/ml creatine phosphokinase (CPK). This assay solution was denoted a60 solution due to its ionic strength of 60 mM. In some other experiments we instead used an assay solution (a45) with ionic strength of 45 mM, achieved by lowering the KCl concentration to 30 mM. Lastly, an oxygen scavenger mixture GOC: (3 mg ml^−1^ glucose, 0.1 mg/ml glucose oxidase, 0.02 mg/ml catalase, final concentrations) was included in the assay solutions for all experiments. HMM aliquots (100 µl) were retrieved from − 80 °C freezer and slowly thawed while buried in ice. Subsequently, the HMM was diluted in wash buffer to its incubation concentration of 120 µg/ml.

Solutions for experiments on the microfluidic platform were like those described above for experiments in conventional flow cells. However, the assay solution a60 was modified to contain additional 2 nM of rhodamine-phalloidin labeled actin filaments.

### Silanization of surfaces for HMM adsorption

Silanization of glass coverslips (Menzel-Gläzer, No. 0; 24 × 60  mm^2^) was performed using trimethylchlorosilane (TMCS), following approaches developed previously^42,58,68^. Molecular sieves (pore size-4Å, diameter − 3.2 mm, Honeywell Fluka, Cat. 334294) were transferred to 3 different glass bottles (1000 ml; 500 ml; 500 ml) in ~ 1/10 (v/v) ratio. Bottles with sieves were placed in an oven (120 °C, 3 h) for evaporation of the remaining water. Subsequently, after removal from the oven, the bottles were kept under a fume hood to cool down for at least 1 h. They were then filled with chloroform (1000 ml and 500 ml) and acetone (500 ml) and kept sealed overnight. On the day of silanization, the solutions were split into several dry glass jars: chloroform (4 jars), acetone (1 jar), methanol (1 jar), approx. 300 ml/jar). Silanization of the glass slides was performed for 10 slides at a time, using a custom-made Teflon holder that was manipulated by modified Pasteur pipettes with hooks made by exposing the pipette ends over a flame. The silanization procedure encompassed the following steps: (i) Incubation in sufficient volume of Piranha solution (95–97% H_2_SO_4_ and 30% H_2_O_2_ in a 7:3 ratio) to cover all the glass slides at 80 °C for 5 min. *Caution: Piranha solution is a highly corrosive and acidic solution that reacts violently with organic materials.* (ii) Incubation in dH_2_O (approx. 800 ml per glass jar) for 2 minutes, 3 times. (iii) Incubation in methanol for 2 minutes. (iv) Incubation in acetone for 2 minutes (v) Incubation in chloroform for 2 minutes. (vi) Drying under a nitrogen gas stream for 1–2 minutes (until sufficiently dry). (vii) Incubation in chloroform with TMCS (5% v/v). Opening and closing the lid to transfer the glass slides in and out of a particular jar was performed under a nitrogen gas stream. (viii) Incubation in chloroform for 2 minutes, 2 times. (ix) Drying under a nitrogen gas stream for 1–2 minutes. The prepared glass slides were stored at room temperature in 100 mm cell culture dishes (2 glass slides per dish) that were sealed with parafilm.

### Degassing and storage of solutions

LISS was the main constituent of all solutions and was therefore carefully degassed. The LISS buffer was first transferred to a flask with a side outlet that connected to an air suction line. The top of the flask was covered with a rubber stopper allowing the creation of low air pressure during suction. Using this approach, 20 ml of the LISS solution was degassed for a minimum of 40 min at 0.7–0.8 bar vacuum under slight mixing using a magnetic stirrer. The prepared buffer solutions were stored on ice in 1.5 ml centrifuge tubes and, as an extra precaution against air entrance, the tubes were wrapped in parafilm. The assay buffer solution was instead transferred to a 1 ml syringe fitted with a hypodermic needle (0.40 × 20 mm, KD-Fine Safety, Germany)) (cf.^38,45^). The syringe was then covered in aluminum foil and the needle-syringe connection was wrapped with parafilm. The syringe was buried in ice until use.

### Assembly of multi-channel flow cells of conventional type

A glass coverslip functionalized with TMCS, as described above, was used as the bottom surface for assembling a flow cell of the type conventionally used for in vitro motility assays. These are the flow cells used here unless otherwise stated. On top of the TMCS-derivatized coverslip, 3 strips of double-sided adhesive tape were placed parallel to each other with a 5 mm gap between neighboring strips. A square coverslip (18 × 18  mm^2^) was then applied to the top of the tape strips, creating 2 flow cells of 10 µl volume. The flow cell openings can be sealed using silicone vacuum grease to prevent oxygen from entering. The latter approach was used only in some experiments in this study. Complete flow cell assembly is illustrated in Fig. 1A−C.

Flow cells for use with a microfluidic pump were assembled as described previously^38^. Briefly, a silanized glass coverslip prepared as above (No. 0; 24 × 60   mm^2^), was used as floor. Above it, an adhesive multi-channel microfluidic slide (sticky-Slide VI 0.4, Ibidi, Cat. No. 80608) was properly secured. After the final addition of assay solution, entry points in one of the channels were sealed by vacuum grease. To the entry points of the channel used for continuous flow of the assay solution, a tubing primed with the same assay solution was attached.

### Microfluidic platform

The microfluidic platform setup is depicted in Fig. S10A. The continuous flow of assay solution was achieved by a Pump 33 Dual Drive System (Harvard Apparatus) to which a 3 ml luer-lock tip syringe (Plastipak, BD) filled with assay solution was secured. The syringe was connected to the microfluidic channel inlet (prepared as described above) with silicone tubing (inner diameter 0.8 mm, Ibidi, Cat. No. 10841) aided with the male elbow luer connector (Ibidi, Cat. No. 10802) on a microfluidic channel side and a needle on the syringe side. To the outlet of the microfluidic channel, similar tubing was attached leading to the waste tube. When connecting the tubing, special attention was dedicated to have it properly primed with the solution so that no air bubble was introduced to the system. A bag of ice was put on the syringe to cool the assay solution during pump action. The flow rate was set to 0.2 µl/ml unless otherwise specified.

### In vitro motility assay procedure using conventional flow cells

The in vitro motility assays were performed following a standard procedure developed from previous work^3,38,55^. First, 20 µl of heavy meromyosin (HMM) at 120 µg/ml (343 nM) was added to each chamber of the flow cell. After incubation for 5 min, 20 µl Bovine Serum Albumin (BSA) solution (1 mg/ml BSA in wash buffer) was added to the chamber and incubated for 2 min. Before the addition of actin filaments, each chamber was washed with 20 µl wash buffer. Then, 20 µl of Rhodamine phalloidin labeled actin filaments (10 nM prepared in wash buffer) were added and incubated for 2 min. Finally, another wash step took place, followed by addition of 2–3 drops of assay solution (from a syringe; see above). A few extra drops of assay solution were added to both sides of the flow cell openings to reduce the risk of drying (Fig. 1C). The latter procedure was not used in the limited number of experiments where the flow cell openings were sealed (Fig. 1B) with silicon vacuum grease (Dow Corning^®^ high-vacuum silicone grease, ref. number Z273554, now Molykote, Dupont). Importantly, the HMM used in the experiments was of sufficient quality, to not require an affinity purification step (ultracentrifugation with actin filaments in the presence of millimolar MgATP) for removal of rigor like (“dead”) heads before application to the flow cell^55^. Neither was a step with blocking actin (non-fluorescent actin filaments added to flow cell at near micromolar concentration) used to block dead heads^55^. Despite the lack of these refinements, the fraction of motile filaments ranged between 0.7 and 1 for all conditions tested.

Unless otherwise stated below, the assay solution was exchanged every 30 min during experiments lasting for up to 8 h. Each exchange of assay solution was associated with addition of new actin filaments as follows: After rinsing with 20 µl of wash buffer, once or twice (as specified below), actin filaments were added, followed by 2 min incubation. Then, another wash was performed using wash buffer, and a fresh assay solution was added. In all cases (unless otherwise stated), a drop of assay solution was placed outside each of the flow cell openings to prevent drying (Fig. 1C).

In between observations, the flow cells were kept in one of two ways: (i) on wet paper to reduce the risk of drying and covered with a lid (a small box for tips wrapped in aluminum foil) at 20–22 °C (room temperature) (Fig. S1). (ii) on the microscope stage in contact with the temperature-controlled objective under a lid (for further details, see under Results).

### In vitro motility assays using the microfluidics platform

The procedure was modified from that for conventional flow cells as follows. The volume of solutions added/aspirated to the flow cells during the different incubation steps to prepare for a motility assay (with HMM etc.) was 40 µl. The pipetting procedure to ensure a complete solution exchange in the microfluidic channel follows the manufacturer (Ibidi) manuals particularly the video protocols for sticky-Slide VI 0.4 (obtained at https://ibidi.com/sticky-slides/65-sticky-slide-vi-04.html). Briefly, the solution additions were done by pipetting the solution against the inner inlet wall (closer to the chamber) while the solution removal was done by placing the tip against the outer wall of the inlet (away from the chamber). For sealed conditions, after the final addition of assay solution to the microfluidic channel, the inlets were filled with an extra 40 µl of assay solution on both sides followed by their sealing with vacuum grease. For continuous flow conditions, the inlets were filled with assay solution following careful attachment to the primed silicone tubing. The latter tubing was further connected with the syringe on the pump for the inlet and with the waste container for the outlet. The first recordings (0 h) were performed 2–3 min after the addition of assay solution and then after approx. every hour till 8 h. In between observations, the microfluidic slide was kept on the microscope stage in contact with the temperature-controlled objective in the empty channel between sealed and continuous flow conditions channels (see also Fig. S10A). Qualitative analysis of motility was performed for each time point (see Fig. S10B, C) and for time points 0, 4, 7 and 8 h we quantified the quality of motility by determining filament velocities and fraction of motile filaments as decried below.

### Imaging, filament tracking and filament analysis

The myosin propelled actin filaments were observed using an inverted fluorescence microscope (Axio Observer.D1 from Zeiss) with a 63 x (Apochromat, NA = 1.4, WD = 0.19 mm) oil immersion objective. The rhodamine phalloidin was excited by a short-arc mercury lamp (103 W/2, from OSRAM) using the fluorescence filter set denoted as Cy3 (Exc: 545/25nm, Di: 565 nm, Em: 605/70nm). Later the mercury lamp was permanently replaced with a LED light source (Colibri 5, Zeiss; used in all experiments with the microfluidics platform). Imaging was performed using an electron multiplying charged-coupled device (EMCCD) camera (C9100-12PHX1, from Hamamatsu photonics). Videos were acquired at 4.95 frames per second (FPS), at a pixel size of 0.24 × 0.24  µm^2^ for a 63x objective. Three videos were acquired, starting 1.5-2 minutes after completed exchange of assay solution. Using a ring-shaped temperature objective heater (Objective Heater 2000, Pecon), connected to a temperature controller (TempController 2000-2, Pecon), a temperature of 24–26 °C was maintained (unless otherwise stated) on the flow cells when motility was observed in the in vitro motility assay. Temperature was measured from a water droplet placed on top of the flow cell, using a 51 II Handheld Digital Probe Thermometer (Fluke). The fraction of motile filaments was estimated from data based on manually counting the total number of filaments and the total number of non-motile filaments in each given video. If needed, the contrast in images/videos was adjusted using the ImageJ function (Image/Adjust/Brightness/Contrast). The sliding velocity was estimated by manually following the leading or trailing end of each filament using a Matlab (Mathworks, Natick, Ma) routine developed previously^16,48^.

### Estimates of fluid evaporation from conventional flow cells

Fluid evaporation from flow cells over time, was estimated from the change in weight of flow cell assemblies with the flow cells filled in the standard way (Fig. 1C) including droplets outside the openings (but using MilliQ water instead of assay solution). The weighing of the flow cell assemblies at different time points was performed using an analytic balance (ME205, Mettler Toledo).

### Estimates of changes in pH in open flow cells

Changes in pH of standard assay solution with time were estimated using a dedicated set of 5 flow cell assemblies (0, 30, 60, 90 and 120 min), filled in the standard way with assay solution (Fig. 1C). Each of the assemblies/flow cells was used to estimate pH after a given storage time. In two experiments pH was alternatively estimated at the end of motility assays in the flow cells used for these assays. The pH was estimated using pH indicator paper (pH-Box, ref. number 1095650001, from Merck, with three paper rolls: blue roll is corresponding to pH range 0.5-5.0 (ACILIT™), yellow to pH range 5.5-9.0 (NEUTRALIT™), and orange to pH range 9.5–13.0 (ALKALIT™), see also Figs. S3, S4—pictures of pH paper). Fluid from the flow cells was withdrawn by bringing the pH paper into contact with the droplet outside the flow cell. A similar procedure was used for sealed flow cells, except that the vacuum grease needed to be carefully removed and/or the glass coverslip roof needed to be broken in order to reach the small amount of liquid inside the chamber.

### Oxygen concentration measurements

The measurements were performed by a multi-parameter meter (Multi 3620 IDS, WTW) attached to the dissolved oxygen probe (FDO 925, WTW) in a 50 ml tube filled with 20 ml of MilliQ water. The volume of MilliQ water for degassing (20 ml) mirrors the typical volume of major assay buffer (LISS) degassed on a typical experimental day using a conical flask with a side outlet connected to the air suction line, placed on a magnetic stirrer plate for slow stirring of the solution during degassing^38^.

### Statistical analysis and reproducibility

No sample size calculations were performed prior to the experiments as the main purpose was not to demonstrate differences in observed variables between protein population groups or from a given population value. Instead, the aim was to test the effect of long-time incubation and solution exchange including information of the variability.

Both velocity data and the fraction of motile filaments were assumed to be normally distributed, consistent with results of the Shapiro-Wilks test, showing no significant difference from the normal distribution (*p* > 0.05). Moreover, a priori, each filament and each observed region of interest on a flow cell was assumed to represent an independent random sample for the velocity and fraction of motile filaments, respectively. This is consistent with previous findings ^11,55,61,69^ and was valid also in the present experiments in cases were statistical hypothesis test were performed (Fig. 5; Figs. S7–S9). The data are presented as mean ± 95% confidence interval (CI) and non-overlapping such confidence intervals are assumed to indicate statistically significant differences between groups (p < ∼0.05). All statistical analyses and data visualization were performed using Graph Pad Prism v. 9.2.0 (Graph Pad Software, San Diego, CA).

## Electronic supplementary material

Below is the link to the electronic supplementary material.

Supplementary Material 1

Supplementary Material 2

Supplementary Material 3

Supplementary Material 4

Supplementary Material 5

## Data Availability

The datasets generated during and/or analysed during the current study are available in the manuscript, the Supplementary information or from the corresponding author upon reasonable request.
