# Supplementary material for: Improved longevity of actomyosin in vitro motility assays for sustainable lab-on-a-chip applications
Source: Sci Rep. 2024 Oct 1;14:22768. doi: 10.1038/s41598-024-73457-x (PMC11445438; doi:10.1038/s41598-024-73457-x)
Supplement: Supplementary file 5 — Supplementary Material 5 [file 41598_2024_73457_MOESM5_ESM.docx]

Supporting Information

Improved longevity of actomyosin in vitro motility assays for sustainable lab-on-a-chip applications

by

Andreas Melbacke^1^, Aseem Salhotra^1^, Marko Ušaj^1^*, Alf Månsson^1^*

^1^Department of Chemistry and Biomedical Sciences, Linnaeus University, SE39182 Kalmar, Sweden

**Supporting Results**

**Effects of evaporation from conventional flow cells and how to limit them**

When using open flow cells it was found essential to apply droplets at the flow cell entrances to avoid drying up of the flow cell interior under prolonged observation/storage. However, the approach has the disadvantage that it increases the liquid-air interfacial area, with increased evaporation that would cause increased ionic strength of the assay solution. To assess the evaporation effect, we weighed the flow cells before and after a 30 min incubation period at 20.7 ^o^C, testing three conditions. Either i) the flow cell was stored on a wet paper under a lid (Fig. S1) to preserve a humid atmosphere, or ii) the lid and the wet paper were omitted or, finally, iii) only the wet paper was omitted. Flow cell weights under these conditions showed that only 3.46 ± 1.08 % (mean ± 95 % CI; N = 5) of the fluid volume evaporated during 30 min in the presence of both wet paper and the lid. If the wet paper was removed but the lid was kept, the evaporation increased to 37.46 ± 3.79 % (N = 5). The degree of evaporation was similar (31.99 ± 3.41 %; N = 5) if also the lid was removed. This analysis demonstrates that a marked increase in ionic strength would occur due to evaporation (e.g., from 60 mM to almost 100 mM with 37 % evaporation) if the flow cell is stored in between subsequent observations without precautions (lid and wet paper). Clearly, this effect would be even higher at higher temperatures, such as 25-30 ^o^C, often used in the in vitro motility assay. The effect may lead to the detachment of filaments from the flow cell surface in the absence of a crowding agent (i.e. methylcellulose), due to weakened electrostatic interactions between actin and myosin, if ionic strength increases above 60-80 mM ^1, 2^. Whereas addition of methylcellulose to the assay solution would eliminate the effect, the use of this viscous additive is inconvenient for solution exchanges. Furthermore, methylcellulose cannot be used in certain nanotechnological applications of the motors because its presence compromises the desired motility contrast between silanized nanochannels and surrounding polymer resist areas ^3, 4^.

**Supporting Figures**

**
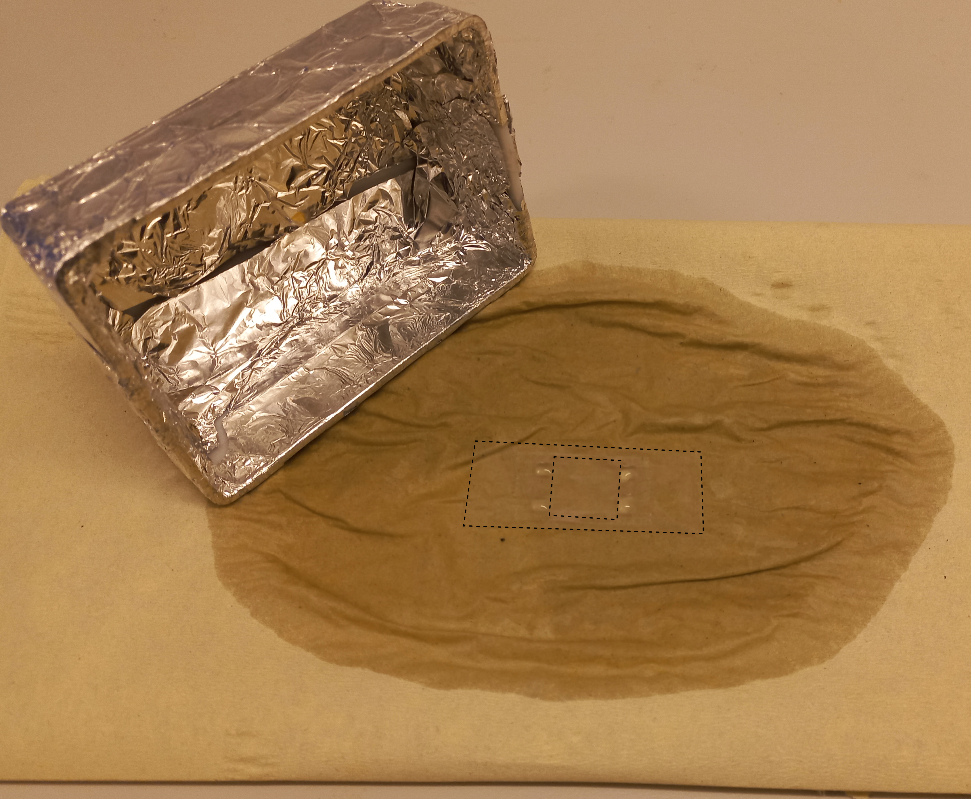
**

**Fig. S1. Wet paper with lid for storage of flow cells to mitigate evaporation.** The plastic lid is covered with aluminum foil (to the left). An assembled flow cell with water droplets outside flow cell openings. The roof and floor coverslips of the flow cell assembly are indicated by dashed lines.

**Fig. S2. Data underlying ratios plotted in Fig. 2 (main paper).** **A.** Sliding velocity vs time after adding assay solution with the flow cell sealed (orange) or open (black). Each data point (mean velocity from 15-18 filaments) represents an independent experiment as defined in main paper. Data from a given experimental day connected by solid lines. **B.** Fraction of motile filaments vs time for the same data as in A, either sealed (orange) or open (black). Each data point represents an independent experiment. Data from a given experiment connected by solid lines.


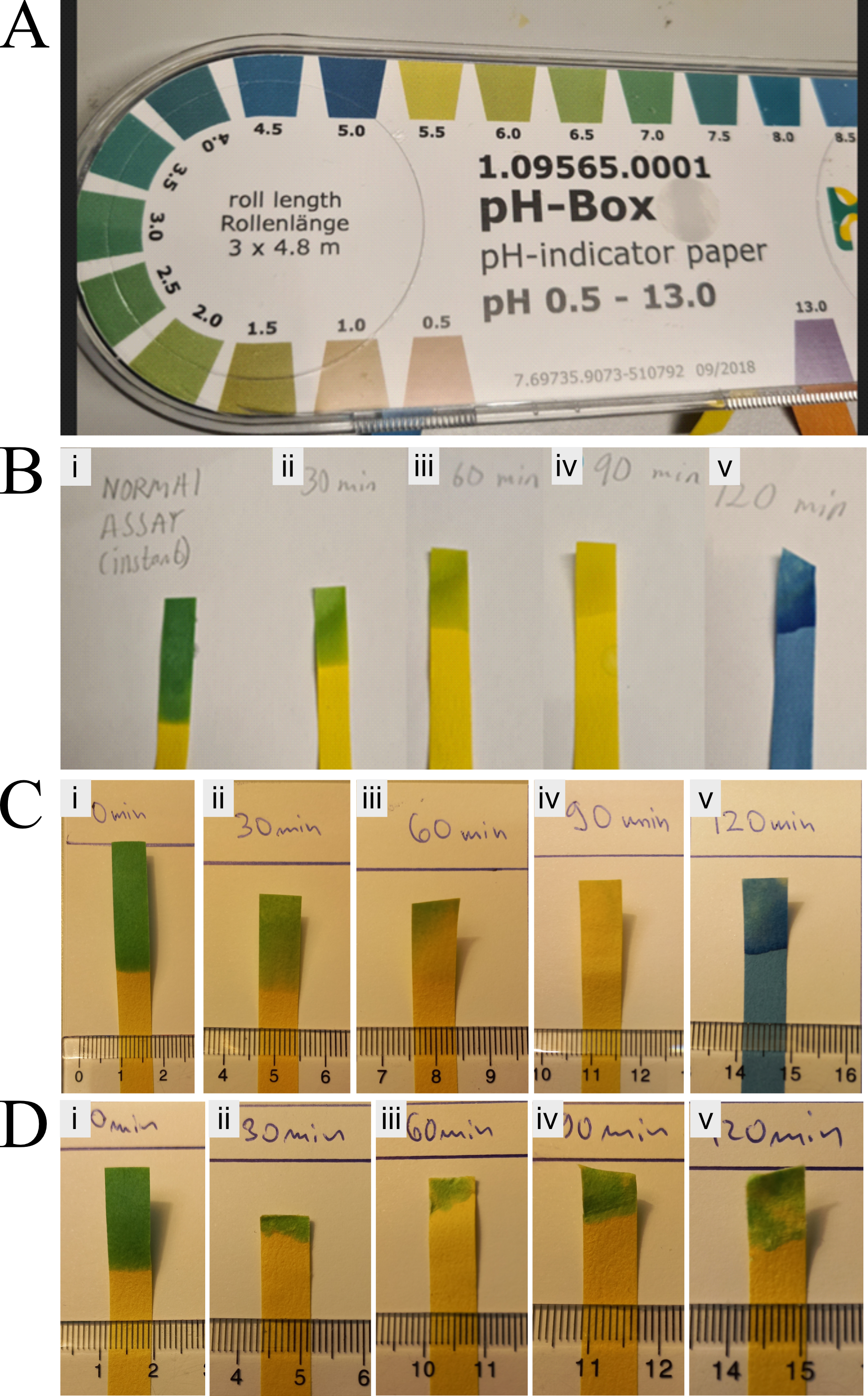


**Fig S3. Use of pH indicator paper to estimate pH in flow cells after storage for different time periods from 0 to 120 min.**

**A.** Gradient used to determine pH**.** Yellow pH-indicator paper was used for pH ranges 5.5-9.0 (NEUTRALIT™; top right) and blue pH-indicator paper was used for ranges 0.5-5.0 (ACILIT™; left). **B.** pH indicator paper appearances, after being subjected to assay solution in standard *open* flow cells at different time points (i-v). i: 0 min, ii: 30 min, iii: 60 min, iv: 90 min, v:120 min. **C.** Repetition of B which was run in parallel with D. **D.** pH indicator paper appearances, after being subjected to assay solution in standard *sealed* flow cells at different time points (i-v). i: 0 min, ii: 30 min, iii: 60 min, iv: 90 min, v:120 min. Note improved pH stability in sealed flow cells. Ruler in C and D in cm.

**
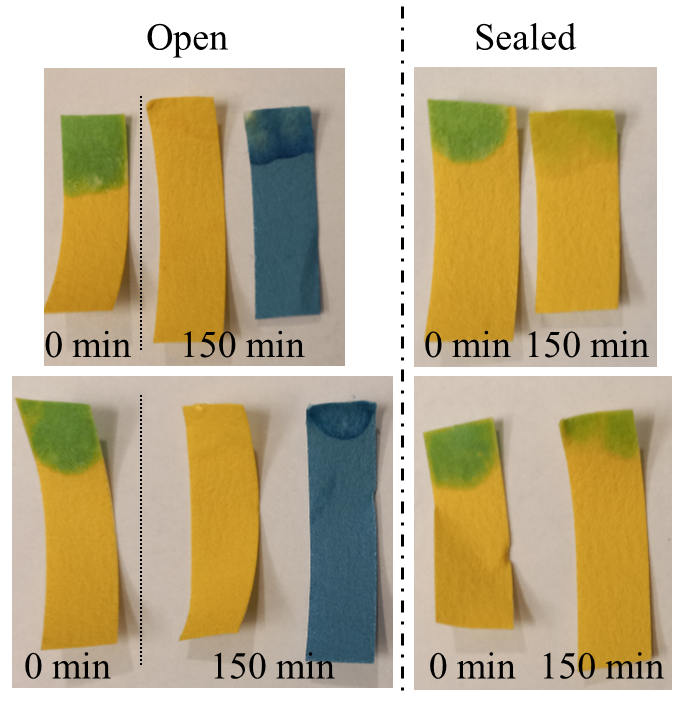
**

**Fig S4. Use of pH indicator paper (as in Fig. S3) to estimate pH in flow cells for additional experiments in Fig. 2 of main paper at 0 and 150 min.** pH estimations for two independent experimental days (*up, bottom*) are shown. Note improved pH stability in sealed flow cells. Gradient used to determine pH was the same as in Fig. S3A (yellow pH-indicator paper was used for pH ranges 5.5-9.0 (NEUTRALIT™) and blue pH-indicator paper was used for ranges 0.5-5.0 (ACILIT™). Please refer to Fig. S3 for more details.

**Fig S5. Degassing efficiency, and re-oxygenation of 20 ml MilliQ water in our laboratory settings. A.** 40 min degassing at -0.8 bar vacuum followed by 1 h on ice in a closed 50 ml tube resulting in ~51 % re-oxygenation (blue triangles). **B.** 60 min degassing at -0.8 bar vacuum followed by 2 h at room temperature in a closed, parafilm-sealed 50 ml tube filled with N2 resulted in ~18% re-oxygenation (inverse green triangles). The data points represent averages of three measurements (n=3) performed on different days. Data given with 95% CI.

**Fig. S6. Individual data points underlying data for a60 protocol in main Fig. 5.** **A.** Sliding velocity in two different experiments (two different flow cells) indicated by filled and open circles, respectively and separated by a vertical dashed line. **B.** The fraction of motile filaments for the two experiments in A. Solution exchange every 30 min. Data given as mean ± 95 % CI for each time point based on 15 actin filaments in A, and observation of 3 regions of interest in B. In these experiments, the flow cells were stored on wet paper under a lid in between motility assays and exchanges, i.e. conditions with expected minimal evaporation during the 3-min storage periods (see above). Furthermore, the decline in pH inside the cell, between observations in the in vitro motility assay, is expected to be too small (Fig. 2 in the main paper) to have any appreciable effects on motility.

**Fig. S7. Minimal difference between sliding velocity (A) and the fraction of motile filaments (B) while using assay solutions with 45 mM (a45) and 60 mM (a60) ionic strength as indicated by the observation of 15 filaments in each solution in A and three regions of interest in the respective flow cell in B**. Data are shown with mean ± 95 % CI.

**Fig. S8. Individual data points underlying data for a45i protocol in main Fig. 5. A**. Sliding velocity in three different experiments (three different flow cells) indicated by filled circles, open circles and diamonds, respectively, as separated by dashed lines. **B**. The fraction of motile filaments for the three experiments in A. Solution exchange every 30 min as described in methods using a45 assay solution. Data are given as mean ± 95 % CI for 15 actin filaments per condition in A, and 3 regions of interests in B

**Fig. S9. Individual data points underlying data for a45ii protocol in main Fig. 5 A.** Sliding velocity in one experiment (one flow cell) over an 8h period. **B.** The fraction of motile filaments for the experiment in A. Solution exchange every 30 min as described in methods using a45 assay solution. Data are given as mean ± 95 % CI. N=15 filaments for each time point in A and N=3 regions of interest for each time point in B.


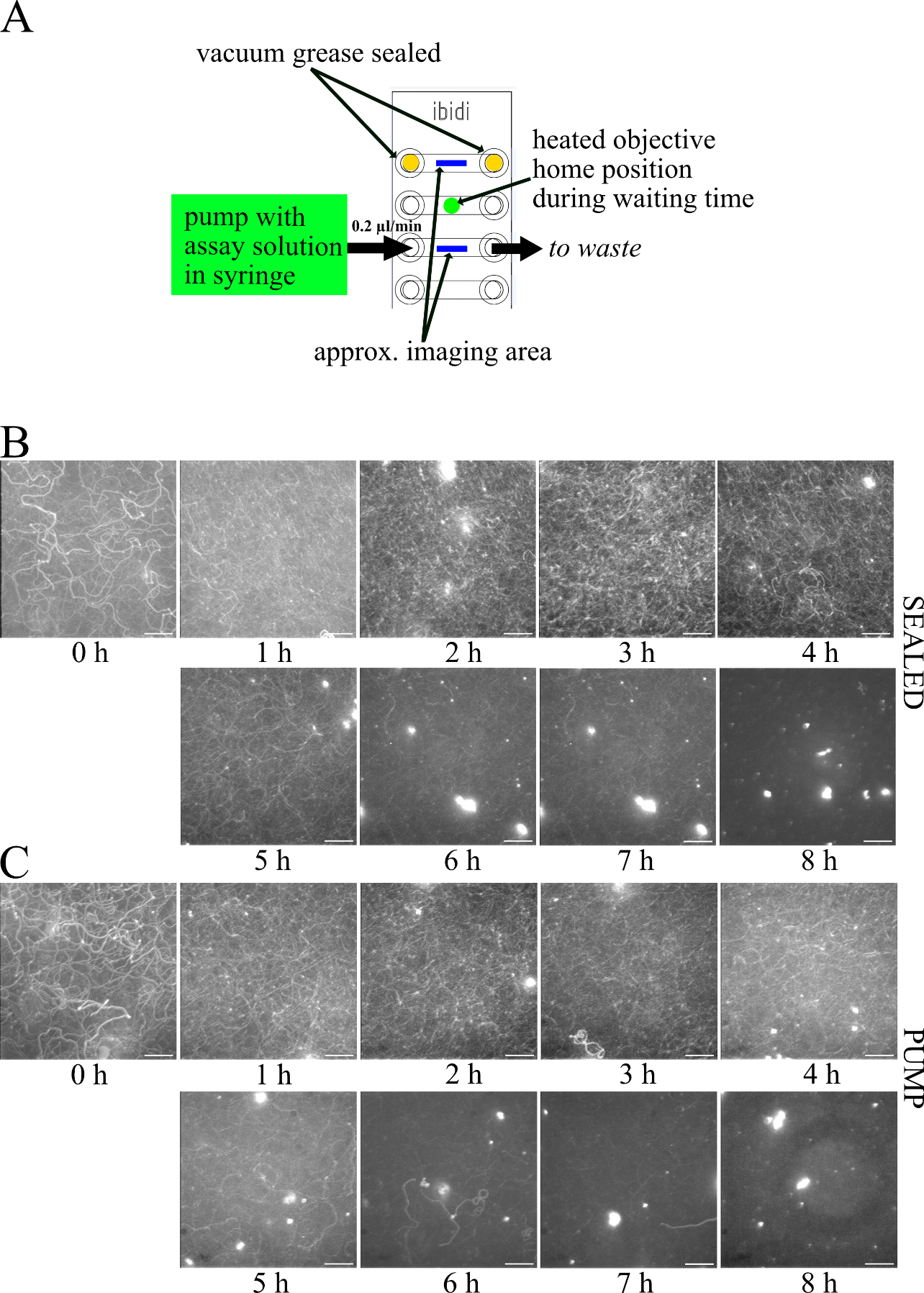


**Fig. S10.** IVMA on a microfluidic platform. **A.** Schematic of the setup. Sketch of an IBIDI slide adapted from a product manual (obtained at [www.ibid.com](http://www.ibid.com)). **B. and C.** Summed maximum intensity projection (Z-projection in ImageJ) of 100 consecutive grayscale images of Rhodamine phalloidin labelled actin filaments captured at 5 FPS at approx. times indicated after the addition of assay solution. *B:* sealed chamber, *C:* chamber under continuous flow of assay solution. Note decreased quality of the signal with prolonged run time. Image intensities adjusted for clarity (ImageJ). Scale bars, 20 µm.

**Temperature dependence of velocity and fraction of motile filaments**

In our first attempt to set in vitro motility assay temperature and storage temperature equal (a45i in Fig. 5; Fig. S8), we kept the flow cell on the microscope stage without using the objective-based temperature control to keep the temperature constant at room temperature. Unfortunately, this approach led to an appreciable increase in velocity and an increase in the fraction of motile filaments over the first 4 h (Fig. 5, S8). We attribute this effect to an increase in temperature with time (see legend of Fig. 5), due to switched-on instruments and heat production from other sources. This idea was confirmed in one separate experiment showing a monotonous increase in temperature from 21 ^o^C at start of the experiment to 25.1 ^o^C after 3.5 h. The latter experiment contains several pieces of valuable information in itself and is therefore considered in further detail here.

In a second sub-protocol (a45ii in Fig. 5; Fig. S9), we kept the temperature constant at 24-26 ^o^C throughout the experiment with the flow cell on the microscope stage throughout. This approach, like the above sub-protocol (a45i), was not compatible with storage on a wet paper to reduce evaporation. The results of the experiment are superimposed on data for the other protocols in Fig. 5 and shown in greater detail in Fig. S9. Clearly, a tendency for an initial increase in velocity and fraction of motile filaments was appreciably lower than in the sub-protocol a45i consistent with a constant temperature in the range 24.5 – 25.5 (measured on top of flow cell).

In a separate experiment we recorded velocity at each temperature in each experiment. We could therefore assess the detailed temperature dependence of motility in the temperature range 21-25.5 ^o^C (Fig. S11). The key finding here was that the temperature sensitivity of velocity greatly declines at temperatures higher than 24 ^o^C. Accordingly, based on an Arrhenius plot of the data, two different Arrhenius activation energies were found, below and above 24 ^o^C, corresponding to two different Q_10_ values of 13.25 and 1.65, respectively. The results can be compared to previous data for fast mammalian muscle fibers ^5^ giving different slopes and Q_10_ values of 2.39 and 1.79 below and above 23 ^o^C, respectively. The results can also be compared to data in ^6^ using a single fiber in vitro motility assay with myosin extracted from fast fibers, showing a breakpoint in the Arrhenius plot at 25 ^o^C, with lower temperature sensitivity of the velocity at the higher (Q_10_ = 1.67) than the lower temperature range (Q_10_ = 2.38). Finally, other studies have shown breakpoints at lower temperatures around 20 ^o^C (cf. ^1^ and references therein). It seems that our results are in good agreement with data from the mentioned studies when it comes to the high temperature range but the temperature sensitivity below 24 ^o^C is appreciably higher in our experiment than in the cited studies. Interestingly, this effect was associated with an unexpected, and quite appreciable, temperature dependence of the fraction of motile filaments in the low temperature range with a notable increase in this fraction with increased temperature (Fig. S11B).

The high temperature sensitivity (Q_10_ ≈ 13) of velocity below 24 ^o^C, in parallel with an increased fraction of motile filaments with increased temperature, is of interest to consider for different reasons. First, it highlights the possibility that other factors than the myosin-actin interaction per se contribute to an apparent temperature dependence of the velocity. It is of interest to note, in this connection, that a high temperature dependence in the in vitro motility assay with a Q_10_ value of >6 was reported for the range 15 – 25 ^o^C also in ^1^.

One possible reason for the high temperature dependence could be different mechanisms of surface adsorption of the motors at different temperatures, i.e. the mode of surface adsorption of already adsorbed heavy meromyosin may change with time if temperature changes. This idea is in accordance with observations of different proportions of myosin with high and low ATP turnover rates at 25 and 15 ^o^C, following the adsorption of heavy meromyosin on TMCS-derivatized surfaces ^7^. Another possibility is that the HMM surface adsorption mechanism changes with time, independent of temperature. In our experiments, where temperature increased with time, this effect would appear to contribute to an apparent temperature dependence. Our observations and the above discussion, add to previous words of caution (^1, 8, 9^) against exclusively interpreting in vitro motility assay results as reflecting physiological mechanisms. However, importantly, when temperature was carefully controlled, the changes with time were small in our experiments.

**Fig. S11 Temperature dependence of the sliding velocity from the temperature changes with time in one experiment using the a45i protocol. A.** Sliding velocity vs temperature. **B.** Fraction of motile filaments vs temperature. **C.** Arrhenius plot of temperature revealing a breakpoint with one linear plot below and one above (to the left in this plot) 24 ^o^C. Data given as mean ± 95 % CI (N=15 for each point in A and C and N=3 for each point in B). Dashed vertical line at 24 ^o^C in all panels.

**Caption of Supporting movies**

**Movie S1 In vitro motility assay just after first addition of assay solution in a45ii protocol (related to Fig. 5 and Fig. S9, first check).** Video shown in real time. Size: 123 x 123 µm^2^.

**Movie S2 In vitro motility assay 4h after first addition of assay solution in a45ii protocol (related to Fig. 5 and Fig. S9).** Video shown in real time. Size: 123 x 123 µm^2^.

**Movie S3 In vitro motility assay 8h after first addition of assay solution in a45ii protocol (related to Fig. 5 and Fig. S9).** Video shown in real time. Size: 123 x 123 µm^2^.

**Movie S4** **In vitro motility assays on the microfluidic platform with sealed (top) and pump (bottom) conditions.** Recordings at ~ 0, 4, 7, and 8 h after addition of the assay solution (related to Fig. 6 and Fig. S10). Videos were accelerated 4 times (from ~5 to 20 fps). Bar represents 20 µm.

**Supporting References**

1. Homsher, E., Wang, F. & Sellers, J.R. Factors affecting movement of F-actin filaments propelled by skeletal muscle heavy meromyosin. *Am. J. Physiol.* **262**, C714-723. (1992).

2. Vikhorev, P.G., Vikhoreva, N.N. & Mansson, A. Bending flexibility of actin filaments during motor-induced sliding. *Biophys. J.* **95**, 5809-5819 (2008).

3. Sundberg, M. et al. Actin filament guidance on a chip: toward high-throughput assays and lab-on-a-chip applications. *Langmuir* **22**, 7286-7295 (2006).

4. Lindberg, F.W. et al. Controlled Surface Silanization for Actin-Myosin Based Nanodevices and Biocompatibility of New Polymer Resists. *Langmuir* **34**, 8777-8784 (2018).

5. Ranatunga, K.W. The force-velocity relation of rat fast- and slow-twitch muscles examined at different temperatures. *J. Physiol. (Lond).* **351**, 517-529 (1984).

6. Hook, P. & Larsson, L. Actomyosin interactions in a novel single muscle fiber in vitro motility assay. *J. Muscle Res. Cell Motil.* **21**, 357-365. (2000).

7. Balaz, M., Sundberg, M., Persson, M., Kvassman, J. & Månsson, A. Effects of Surface Adsorption on Catalytic Activity of Heavy Meromyosin Studied using Fluorescent ATP Analogue. *Biochemistry.* **46**, 7233-7251 (2007).

8. Rahman, M.A., Salhotra, A. & Mansson, A. Comparative analysis of widely used methods to remove nonfunctional myosin heads for the in vitro motility assay. *J. Muscle Res. Cell Motil.* **39**, 175-187 (2018).

9. Sugi, H. et al. Definite differences between in vitro actin-myosin sliding and muscle contraction as revealed using antibodies to myosin head. *PLoS One* **9**, e93272 (2014).
